# Hydration of Heavy Alkaline-Earth Cations Studied by Molecular Dynamics Simulations and X-ray Absorption Spectroscopy

**DOI:** 10.1021/acs.inorgchem.1c01888

**Published:** 2021-08-13

**Authors:** Rafael R. Pappalardo, Daniel Z. Caralampio, José M. Martínez, Enrique Sánchez Marcos

**Affiliations:** Department of Physical Chemistry, University of Seville, 41012 Seville, Seville, Spain

## Abstract

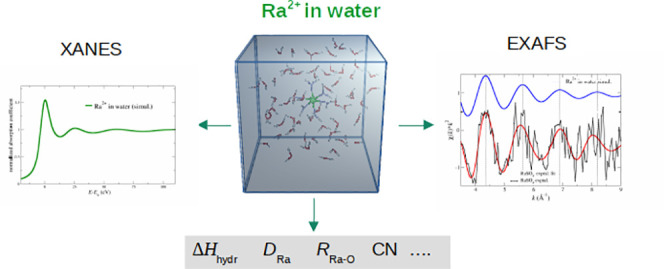

The physicochemical properties of the three heaviest alkaline-earth cations, Sr^2+^, Ba^2+^, and Ra^2+^ in water have been studied by means of classical molecular dynamics (MD) simulations. A specific set of cation–water intermolecular potentials based on ab initio potential energy surfaces has been built on the basis of the hydrated ion concept. The polarizable and flexible model of water MCDHO2 was adopted. The theoretical–experimental comparison of structural, dynamical, energetic, and spectroscopical properties of Sr^2+^ and Ba^2+^ aqueous solutions is satisfactory, which supports the methodology developed. This good behavior allows a reasonable reliability for the predicted Ra^2+^ physicochemical data not experimentally determined yet. Simulated extended X-ray absorption fine-structure (EXAFS) and X-ray absorption near-edge spectroscopy spectra have been computed from the snapshots of the MD simulations and compared with the experimental information available for Sr^2+^ and Ba^2+^. For the Ra^2+^ case, the Ra L_3_-edge EXAFS spectrum is proposed. Structural and dynamical properties of the aqua ions for the three cations have been obtained and analyzed. Along the [M(H_2_O)_*n*_]^*m*+^ series, the M–O distance for the first-hydration shell is 2.57, 2.81, and 2.93 Å for Sr^2+^, Ba^2+^, and Ra^2+^, respectively. The hydration number also increases when one is going down along the group: 8.1, 9.4, and 9.8 for Sr^2+^, Ba^2+^, and Ra^2+^, respectively. Whereas [Sr(H_2_O)_8_]^2+^ is a typical aqua ion with a well-defined structure, the Ba^2+^ and Ra^2+^ hydration provides a picture exhibiting an average between the ennea- and the deca-hydration. These results show a similar chemical behavior of Ba^2+^ and Ra^2+^ aqueous solutions and support experimental studies on the removal of Ra-226 of aquifers by different techniques, where Ra^2+^ is replaced by Ba^2+^. A comparison of the heavy alkaline ions, Rb^+^ and Cs^+^, with the heavy alkaline-earth ions is made.

## Abstract 2

A force field to describe Ra^2+^ in water based on a QM potential energy surface has been built and applied via classical MD simulations to predict physicochemical properties in solution such as hydration enthalpy, diffusion coefficient, or XAS spectra. To support the reliability of the methodology, Sr^2+^ and Ba^2+^ force fields have also been developed to assess the procedure in the case of the much more well-known solution chemistry of these heavy alkaline-earth cations.

## Introduction

The solution chemistry of alkaline-earth cations is extremely wide, being involved in a huge number of domains of chemistry, biology, geology, and chemical engineering.^1−3^ Whereas the lighter alkaline-earth cations, particularly, Mg^2+^ and Ca^2+^, are ubiquitous in many systems and natural environments, having been deeply studied by experimental and theoretical techniques, their heavy alkaline-earth group companions are much less disseminated and their solution chemistry is more limited, particularly that of Ra^2+^. The mitic radium discovery by Marie and Pierre Curie opened the field of the nuclear chemistry,^4^ but both its extremely low abundance in nature and its very high radioactivity that makes its experimental handling hazardous have limited its data compilation. Thus, there are not experimental studies providing the hydration number nor the average Ra–O(H_2_O) distance of the aqua ion. These shortcomings stimulate the use of theoretical tools to undertake the estimation of physicochemical properties hardly accessible by experiments.^5,6^ The problem arising from this strategy is to evaluate the degree of confidence of these theoretical results if experimental results are not available for comparison. Two precedent alkaline-earth cations, Sr^2+^ and Ba^2+^, are good candidates to be investigated together with Ra^2+^ in order to analyze the evolution of properties along the group in a systematic way, starting from a regular increase of the ionic radius. Luckily, for these two ions, there is enough experimental chemical information to validate the theoretical results.

There are several works about the structural properties of the Sr^2+^ aqua ion. By means of extended X-ray absorption fine structure (EXAFS) studies,^7−13^ coordination numbers between 7.3 and 10.3 have been proposed, 8 being the most usual value.^9,11,14,15^ A small range of Sr–O distances, between 2.57 and 2.64 Å was found. By means of classical molecular dynamics (MD),^14−18^ coordinations of ∼8 have been found with Sr–O distances in the range 2.5–2.7 Å. In a quantum mechanics/molecular mechanics (QM/MM) study,^19^ a coordination number of 9 with the peak distance at 2.69 Å was reported. Data are collected in Table 2.

There are several experimental studies about Ba^2+^ hydration, which are collected in Table 2. Persson et al.^9^ determined a coordination number of 8 with an interatomic Ba–O distance of 2.82 Å by large-angle X-ray scattering–EXAFS. Recently, Migliorati et al.^20^ have studied Ba^2+^ hydration by EXAFS spectroscopy and computer simulations. Their EXAFS fit found a Ba–O distance of 2.85 Å and a hydration number of 8. MD studies^14,18,20,21^ using rigid water models obtained the first-shell coordination numbers between 8 and 8.8 with peak distances in the 2.75–2.85 Å range. Hofer et al.^22^ obtained a first-shell coordination number of 9.3 with the peak distance at 2.86 Å and a second shell formed by ∼24 water molecules by means of a QM/MM study. The later results are in contrast with those derived from an ab initio MD study of Rempe’s group,^23^ where an octa-coordination was found at 2.8 Å.

There are two quantum mechanical studies on the formation of Ra^2+^ hydrated clusters,^5,24^ and only one theoretical work devoted to study its hydration in aqueous solution^6^ by means of the fragment molecular orbital–MD technique. A first-shell coordination number of 8.1 was predicted with a peak distance of 2.85 Å. No experimental characterization of the radium aqua ion has been found in the literature, but an experimental Ra–O distance has been determined by EXAFS by Hedström et al.^25^ for the radium sulfate crystal.

This work aims to carry out a systematic theoretical study on the hydration of the three heaviest alkaline-earth cations. The MD simulations of the three cations in water were performed on the same methodological basis so that the difference found must be a consequence of the intrinsic physicochemical properties of the ions. The comparison of theoretical and experimental results, mainly for Sr^2+^ and Ba^2+^, will assess our methodology and, on the basis of good agreement, support the physicochemical properties found for Ra^2+^ in aqueous solution.

## Methods

This section has been split into three parts, the first of them being devoted to the building of the cation–water intermolecular potentials based on the hydrated ion model proposed by our group.^26−28^ The second part gives the QM and MD computational details. The third one describes the procedure to simulate the X-ray absorption spectra.

### Interaction Potentials

The basic idea of the hydrated ion model is the recognition that the representative species interacting in aqueous solution is the charged metal cation surrounded by a given number of water molecules.^1^ Then, our original statistical implementation of the concept was based on the development of a hydrated ion–water interaction potential ([M(H_2_O)_*n*_]^*m*+^–H_2_O) for stable aqua ions based on first-principles.^26,28^ The consideration of the [M(H_2_O)_*n*_]^*m*+^ aggregate as the key entity to deal with intermolecular interactions is a natural way to include many-body effects in an effective pairwise potential. Later on, a second potential was built to describe the intrinsic aqua ion dynamics, that is, the ion–water first-shell (IW1) interaction^27,28^ ([M–(H_2_O)_*n*_]^*m*+^). These potentials have been applied to a set of stable monoatomic^28−31^ and molecular^32,33^ aqua ions, and we refer to these studies for further details. Aqua ions’ stability ranges from few hundreds of years for [Ir(H_2_O)_6_]^3+^ to about few picoseconds for several lanthanoid and heavy alkaline aqua ions.^34^ Our model was only valid when the time of first-shell water release is longer than the simulation time. Otherwise, there would be “first-shell” water molecules in the bulk during the simulation time. To overcome this shortcoming in the methodology, an improvement was added considering that there are not two types of water molecules in the simulation, but water molecules are sensitive to the local environment by means of a polarizable and flexible potential, the mobile charge density harmonic oscillator (MCDHO), which is a shell model.^35^ This exchangeable hydrated ion model has been already applied to the description of aqueous solutions of trivalent lanthanoid and actinoid cations.^36−40^ In the present study, we have chosen the MCDHO2 potential,^41^ a revised MCDHO version which improves the water mobility description, as already proven for the demanding cases of the heavy alkaline Rb(I) and Cs(I).^42^ Details on the functional form and the potential construction are given in Supporting Information.

Figure S2 of Supporting Information displays some representative structures used to build the intermolecular potentials. About 470 structures were used for each of the Sr^2+^–H_2_O and Ba^2+^–H_2_O interaction potential building, whereas only 135 points have been necessary for the Ra^2+^–H_2_O case. The Sr^2+^–H_2_O potential has been built using hydrates with seven, eight, and nine water molecules, as well as hydrates with up to two water molecules in the second shell. The addition of surface clusters with 7–10 water molecules improved the global behavior of the potential. The Ba^2+^–H_2_O potential was built using structures with eight and nine water molecules and up to two water molecules in the second shell. The Ra^2+^–H_2_O potential was built using structures with eight and nine water molecules in the first shell and structures with one or two water molecules in the second shell.

Table S2 in Supporting Information collects the force field parameters for the Sr^2+^–H_2_O, Ba^2+^–H_2_O, and Ra^2+^–H_2_O intermolecular potentials. Table 1 shows a set of interaction energies and M–O distances for several [M(H_2_O)_*n*_]^2+^ clusters computed at the QM level together with the values obtained with the force field (pot). The interaction energy has been calculated as the energy difference between the hydrated cluster energy and the energy, at the minimum-energy geometry, of the isolated fragments, as shown in eq 1
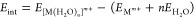
1

**Table 1:** Interaction Energies and M–O Distances of Sr^2+^, Ba^2+^, and Ra^2+^ Hydrates^a^

	*E*_int_ (kcal/mol)			*R*_M–O_ (Å)	
structure	QM//QM	pot//QM	pot//pot	*R*_QM_	*R*_Pot_
Sr(H_2_O)_7_^2+^	–251.3	–254.6	–256.5	2.56	2.55
Sr(H_2_O)_8_^2+^	–272.5	–272.4	–274.8	2.59	2.59
Sr(H_2_O)_8_^2+^(H_2_O)	–292.6	–288.8	–292.5	8 × 2.59/1 × 4.52	8 × 2.59/1 × 4.43
Sr(H_2_O)_9_^2+^	–288.6	–284.1	–288.2	2.62	2.64
Ba(H_2_O)_8_^2+^	–241.2	–241.1	–247.0	2.79	2.77
Ba(H_2_O)_8_^2+^(H_2_O)	–260.9	–258.9	–264.2	8 × 2.79/1 × 4.70	8 × 2.79/1 × 4.59
Ba(H_2_O)_9_^2+^	–258.0	–257.0	–263.2	2.82	2.82
Ra(H_2_O)_8_^2+^	–215.4	–214.2	–225.2	2.91	2.90
Ra(H_2_O)_9_^2+^	–236.6	–235.1	–241.6	2.99	2.94
Ra(H_2_O)_10_^2+^	–255.1	–253.0	–256.8	2.96	2.98

a The following notation is used: “calculation level employed//origin of the geometry used”.

As can be seen, there is a good energy and structural agreement for the most likely coordination numbers in solution. Also, there is a good energy reproduction of the whole set of structures included in the fitting as shown in Figures S3–S5 of Supporting Information, with standard deviations of 3.5, 2.7, and 3.7 kcal/mol, for Sr^2+^, Ba^2+^, and Ra^2+^, respectively.

### MD and QM Computational Details

MD simulations in the canonical ensemble (*NVT*) at 300 K were carried out using an in-house modification of the DL_POLY program^43^ (classic version), which allows the use of the MCDHO2 potential. A fictitious mass of 0.1 a.u. is associated with the mobile charges of the cations and water oxygen in order to solve the atomic and molecular charge distribution by propagating these fictitious masses together with the rest of real particles. Simulation box contained 1 metal cation and 1000 water molecules, with a box length that fits the experimental water density at this temperature (0.997 g/cm^3^). Also, 1 ns was produced for each cation with a time step of 0.1 fs. Long-range interactions were treated by means of the Ewald sum with a cutoff radius of 14 Å and 10^–6^ for relative error.

The QM calculations to develop the intermolecular potentials have been performed at the M06-2X/def2-TZVPP level using the effective core pseudopotentials (ECP) of the Stuttgart group: ECP28MDF^44^ for Sr, ECP46MDF^44^ for Ba, and ECP78MDF^44^ for Ra, including 28, 46, and 78 electrons, respectively, in the core. The eight valence electrons orbitals are described by the corresponding basis sets associated with the ECPs. The Gaussian 09^45^ code was used for the QM calculations.

### Simulated X-ray absorption spectroscopy (XAS) Spectra

The simulated spectra were obtained by averaging the computed spectra of snapshots evenly taken from the MD simulation, as previously done by several authors in other metal ion-in-solution studies.^46−50^ To assess the convergence of the simulated XAS spectra with the average number of snapshots, simulated EXAFS spectra with different number of snapshots were computed. A total of 250 snapshots evenly spaced through the 1 ns trajectories are enough to reach convergence. For each snapshot, the K-edge for Sr, the L_3_-edge for Ba and Ra, EXAFS, and X-ray absorption near-edge spectroscopy (XANES) spectra were computed by means of the FEFF 9.6 code.^51^ This program calculates ab initio EXAFS and XANES spectra by taking into account multiple scattering effects. Sample input files employed to calculate the simulated spectra are given in Supporting Information (see Figures S6 and S7). The inclusion of hydration shells beyond the first one in the snapshots has no effect on the simulated spectra. This was confirmed by the comparison of two sets of simulated spectra, where the cutoff radius around the metal cation was such that in one case, only the first-shell water molecules were included and in the other case, the second hydration shell was also included. The Hedin–Lundqvist exchange–correlation potential was used to compute the electron density distribution within the self-consistent field approach. The inner potential correction, Δ*E*_0_ was chosen such that the simulated spectrum overlaps the experimental one as much as possible.

### Removal of the Multielectron Excitation from Experimental EXAFS Spectra

Experimental EXAFS spectra for Sr^2+^ and Ba^2+^ aqueous solutions present multielectron excitations in the region 5–7 Å^–1^.^13,52^ The identification of multielectron excitations in the EXAFS spectra and their removal is necessary to obtain structural information. Several strategies have been suggested for this purpose.^53−56^ We have adopted the procedure proposed by Ohta et al.,^57^ which was successfully applied in a previous work^42^ to the EXAFS signals of Rb^+^ and Cs^+^. We refer to this paper for further details.

## Results and Discussion

Table 2 collects structural and dynamical properties of the three heavy alkaline-earth cations in water derived from the analysis of the MD simulations. Experimental and theoretical data reported by previous studies have also been included in the table. It must be noted that for Ra^2+^, the available information is very scarce. A global prospect of the results obtained in this study shows that our results are in general within the wide range of the values proposed by precedent studies. The reason of such a wide range is, at least partially, due to the fact that there are a large number of different techniques included in the data collection.

**Table 2:** Properties of Heavy Alkaline-Earth Cation Aqueous Solution^a^

	Sr^2+^		Ba^2+^		Ra^2+^	
property	this work	literature	this work	literature	this work	literature
*R*_M–O__I_(Å)	2.57	2.5–2.64^7−11,13−19,21,61^	2.81	2.60–2.86^9,14,18,20−23,52^	2.93	2.85^6^
CN_I_	8.1	7.3–10.3^7−19,61^	9.4	7.8–9.3^9,14,18,20−23,52^	9.8	8.1^6^
DW (Å^2^)	0.017	0.0116,^9^ 0.0115,^12^ 0.021^13^	0.034	0.0112,^9^ 0.012,^52^ 0.018^20^	0.034	
tilt angle_I_ (deg)	141(19)	155^19^	138(38)	155^22^	135(22)	
ϵ (eccentricity)	0.17(0.08)		0.22(0.10)		0.24(0.10)	
*R*_M__–__O__II_(Å)	4.72	4.78–4.85,^9,61^ 4.97,^19^ 4.9^11^	4.92	4.90,^9^ 5^22^	5.02	
CN_II_	22	19.0–22.7,^61^ 23.5,^19^ 15^11^	20	23.5^22^	20.9	
tilt angle_II_ (deg)	110(38)		111(20)		109(38)	
Δμ_I_ (D)	0.2(0.3)		0.1(0.3)		0.0(0.3)	
Δμ_II_ (D)	0.0(0.3)		0.0(0.3)		0.0(0.3)	
MRT (t* = 0) (ps)	65(4)	∼100–1000_,_^34,62^ 43,^14^ 182^18^	38(4)	∼100–1000,^34,62^ 15,^14^ 148^18^	20(4)	
MRT (t* = 2) (ps)	90(4)		53(4)		29(4)	
τ_1,_μ (ps)	44		26	8.8^22^	29(4)	
τ_2,_μ (ps)	8		4	3.1^22^	4	
Δ*H*_hyd_ (kcal/mol)	-353(15)	–351.3,^2^ −344.9^9^	–322(12)	–318.4 (2), −311.9,^9^ −300^21^	–302(21)	–315.7 (2), −302.6^9^
*D* (10^–5^ cm^2^/s)	0.6(0.2)	0.79,^2^ 0.72,^16^ 0.66^18^	0.8(0.1)	0.847,^2^ 0.9,^14^ 0.64^18^	0.9(0.1)	0.89^2^

a Standard deviation in parenthesis.

The first illustrative property assessing the classical force field employed in the simulation is the hydration energy. The calculated hydration enthalpy follows the experimental trend,^2^ |Δ*H*_hydr_(Sr^2+^)| > |Δ*H*_hydr_(Ba^2+^)| ≈ |Δ*H*_hydr_(Ra^2+^)|, and the worst estimation only deviates 4% from the experimental value. Interestingly, Persson et al.^9^ examined the experimental hydration enthalpy for the alkaline-earth group, from Be^2+^ to Ba^2+^, and found a reasonable linear correlation with the inverse of the M–O distance. Their values for Sr^2+^ (−344.9 kcal/mol) and Ba^2+^ (−311.9 kcal/mol) are almost the same values obtained in this work considering the uncertainties: −353 ± 15 and −322 ± 12 kcal/mol for Sr^2+^ and Ba^2+^, respectively. Their extrapolated hydration enthalpy for Ra^2+^ is −302.6 kcal/mol, the same value predicted by us (see Table 2). Bearing in mind the uncertainties associated with our estimation and the model employed by Persson et al.,^9^ this perfect match must be regarded with caution. Nevertheless, the result reinforces the coherence of the hydration energies derived from the use of the new potentials. The sequence qualitatively follows the size of the hydrated ion, what can be roughly estimated by the first minimum in the M–H radial distribution functions (RDFs): 3.97, 4.27, and 4.30 Å for Sr^2+^, Ba^2+^, and Ra^2+^, respectively. When dealing with small cations, it has long been recognized that their hydration energies can be roughly split into two contributions. The first one is due to the specific interactions of the first-shell water molecules with the central cation and among them. The second contribution is due to the interactions of the hydrated ion with the bulk represented by a polarizable dielectric continuum, which is a generalization of the Born term. This strategy^58−60^ known as the semicontinuum solvation model is an operative way to supply a generally well-balanced description of the energetics of the solvation phenomenon. In our particular case, the aqua ion size is a good structural parameter to estimate the Δ*H*_hydr_ sequence of these heavy alkaline-earth cations. The discrete contribution is dominated by the direct interaction of the first-shell water molecules with the cations whose charge is the same for all of them. The closer the water molecules are to the cation, the stronger the interaction. The minimum of M–H_I_ RDF is a parameter featuring this fact. The sequence of this contribution is also reflected in Table 1, where the interaction energies both from QM calculations and the developed potentials favor Sr^2+^ versus Ba^2+^ and Ra^2+^ aqua ions. For aqua ions with the same charge, the continuum contribution is greater when the cavity size is smaller; therefore, the contribution to the Sr^2+^ hydration will be larger than for Ba^2+^ and Ra^2+^.

When comparing with their alkaline partners, Rb^+^ and Cs^+^, computed at the same level of calculation,^42^ it is observed that their hydration enthalpies are much smaller, −70 and −55 kcal/mol, respectively. This means between 4.5 and 5 times smaller than those of the heavy alkaline-earth cations. A significant part of this gap is due to the cation charge, that according to the Born term has a quadratic dependence. However, there is also a structural factor associated with the hydrated ion size, which is bigger for the monovalent cations; the first minimum M–H_I_ RDF appears at 4.4 and 4.7 Å, for Rb^+^ and Cs^+^, respectively. Therefore, the cavity size also contributes to a smaller Born term.

### Structural Properties

**Figure 1:**
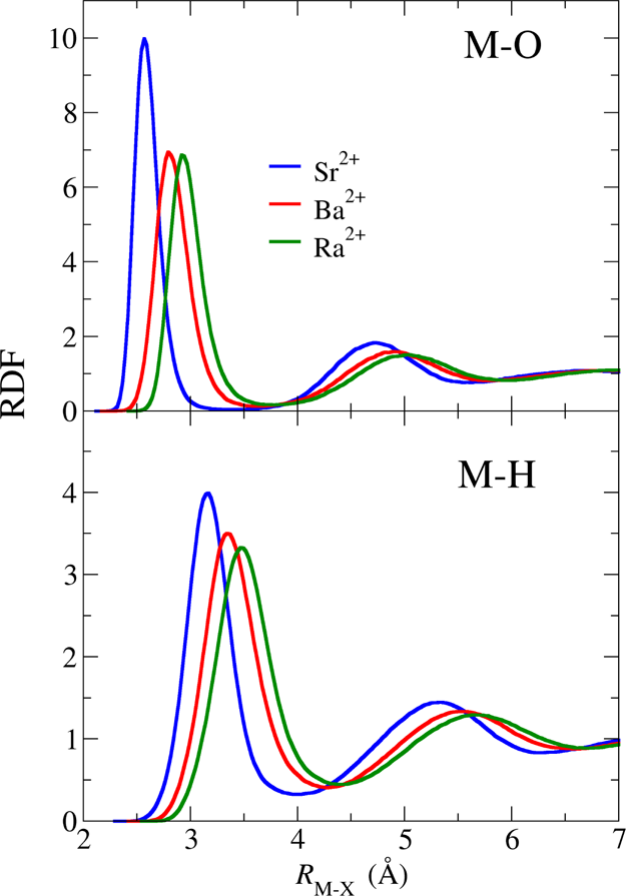
Metal–oxygen (top) and metal–hydrogen radial distribution functions.

Figure 1 plots the M–O and M–H RDFs of the three heavy alkaline-earth divalent cations in water. The first and second hydration shells for M–O are well defined, particularly for Sr^2+^, with a depletion zone between them, indicating the existence of aqua ions in the three cases. Their sizes increase when progressing through the group, as reflected by both the longer M–H_I_ distance when going from Sr^2+^ to Ra^2+^, already mentioned above, and the larger coordination number [CN(M–O_I_) = 8.1 (Sr^2+^), 9.4 (Ba^2+^), and 9.8 (Ra^2+^)]. Regarding the hydration number for Sr^2+^, there is a general agreement about the eightfold coordination from both experimental results^8,9^ and computer simulations.^14^ First-shell Sr–O distances close to our results have also been found in both experimental EXAFS fitting (2.57^8^ and 2.63^9^ Å) and simulations, 2.58 Å.^14,15^ For Ba^2+^, it is observed that the hydration number derived from most of the experimental studies is a value close to 8,^9,20^ whereas statistical simulations predict a wider range of values: 8,^20^ 8.8,^14^ and 9.3.^22^ When we examine the simulated EXAFS spectrum, we will come back to this question. The Ba–O distance for the first-shell found in this work, 2.81 Å, agrees, within the uncertainties, with EXAFS fitting (2.78,^52^ 2.81,^9^ 2.85^20^ Å, and X-ray diffraction data, 2.80^63^ Å). Likewise, the results from statistical simulations are in the range 2.78^14^–2.86^22^ Å, close values to the distance was found in this work.

**Figure 2:**
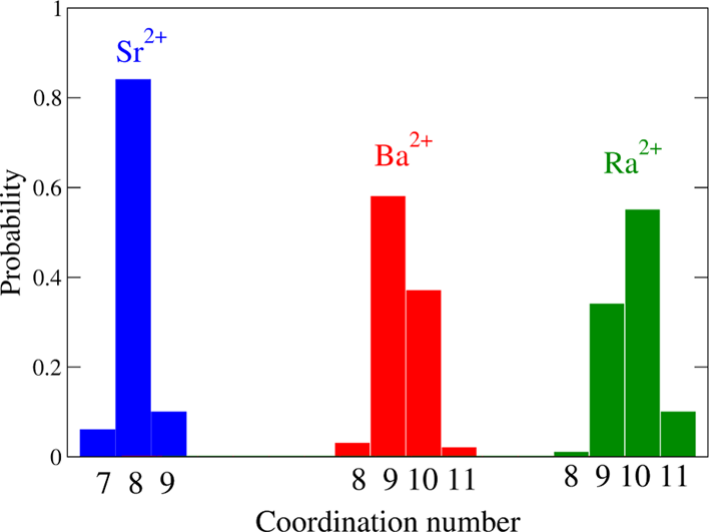
Histogram of the coordination number for the different cation hydrates.

A complementary view of the RDF first-peak is the analysis of the M–O first peak composition by examining the hydrates distribution of different coordination numbers for the three cations obtained during the simulations. Figure 2 shows the probability of a given hydration number during the simulation. The octahydrate is clearly predominant for the Sr^2+^ case, whereas for the Ba^2+^ and Ra^2+^ ions the distribution is mainly shared between the ennea- and deca-coordinations although with different preferences: the ennea-hydrate is dominant in the Ba^2+^ case while for Ra^2+^ is the deca-hydrate. RDFs also reflect the existence of second hydration shells formed in all cases with defined second peaks centered for M–O at 4.73 (Sr^2+^), 4.92 (Ba^2+^), and 5.03 (Ra^2+^) Å. Their running integration numbers account for 22–20 water molecules. In the case of the heavy alkaline partners, the second hydration shell is poorly defined for Rb^+^ and absent for Cs^+^,^42^ and there is no depletion zone between the first and the second hydration shells for neither of them.

Another parameter representative of the aqua ion consistency is the second cumulant of the first shell ion–oxygen distance defined by means of eq 2
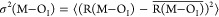
2a parameter which is associated to the Debye–Waller (DW) factor in spectroscopical and diffraction techniques. The value for the Sr^2+^ aqua ion is a half of the values found for the other two cations (see Table 2), Ba^2+^ and Ra^2+^ having similar values for the DW factors.

Inspection of the M–H RDFs leads to an analysis similar to that of the M–O RDFs. The shifting of the first peak of the M–H RDF with respect to that of the M–O RDF indicates that on average, water molecules adopt an ion–dipole orientation with respect to the central cation. The tilt angle of the first-shell water molecules is one of the geometrical parameters quantifying this structural feature. The asymptotic value of 180° means that the water molecule plane contains the cation, that is, the ion–dipole interaction is maximized. The tilt angle does not change too much along the three cases, although it decreases smoothly from 141° for Sr^2+^ to 138° and 135° for Ba^2+^ and Ra^2+^, respectively. These values indicate that theres is a significant cation induced orientation, in spite of thermal effects and water–water interactions. The polarization effects are also reflected in the dipole moment increase experienced by the first-shell water molecules, 0.2 D for Sr^2+^, 0.1 D for Ba^2+^, and no change for Ra^2+^.

An interesting structural parameter representative of the degree of symmetry of the aqua ion is the molecular eccentricity, ϵ.^42^ This is computed as the average distance between the metal cation and the center of mass of the first hydration shell, as shown by eq 3
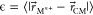
3

In the case of a rigid and symmetric aqua ion, the value of ϵ along the simulation must be 0. A typical value for the Li^+^ tetrahydrate, a consistent aqua ion, is 0.2 Å, whereas for the Cs^+^ hydrate, which has a poorly structured hydration shell, its value is 0.45 Å, the value being 0.37 Å for the intermediate case of the Rb^+^ hydrate.^42^ For the cases of the divalent cations studied here, the Sr^2+^ octahydrate has a value of 0.17 Å, whereas 0.22 and 0.24 Å are the values for the Ba^2+^ and Ra^2+^ ennea- and deca-hydrates, respectively.

### XAS Spectra

**Figure 3:**
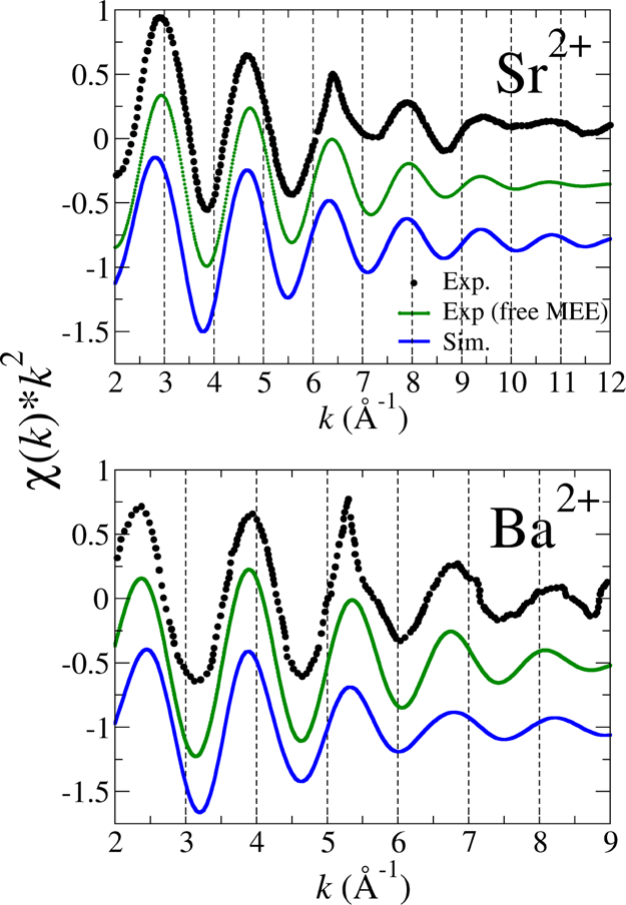
Sr K-edge and Ba L_3_-edge *k*^2^-weighted EXAFS spectra of their Sr^2+^ and Ba^2+^ aqueous solution: experimental spectra taken from ref (9) (black dots), MEE-removed experimental spectrum (green line), and simulated spectrum (blue line).

**Figure 4:**
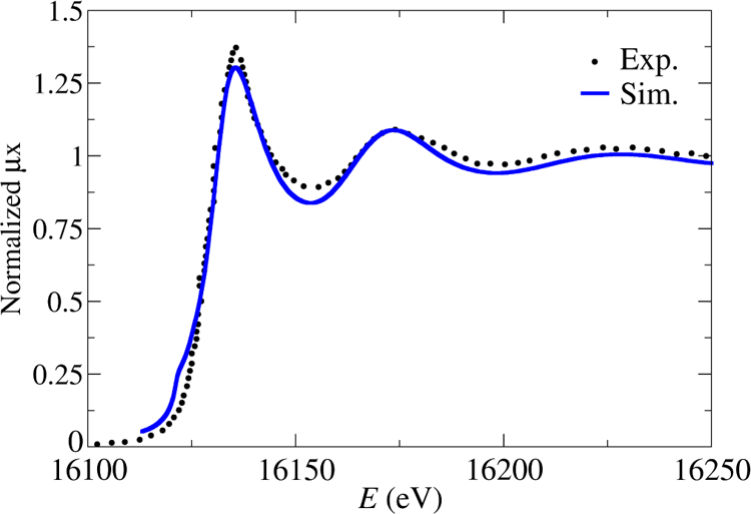
Sr K-edge XANES spectra of a Sr^2+^ aqueous solution, experimental spectrum taken from ref (9) (black dots) and simulated spectrum (blue line).

The comparison of the experimental Sr K-edge and Ba L_3_-edge *k*^2^-weighted EXAFS spectra recorded by Persson et al.,^9^ for 0.1 and 0.2 M aqueous solutions containing strontium and barium triflate, with the simulated spectra is shown in Figure 3. The experimental EXAFS spectra (black dotted lines) present a multielectron excitation^13^ (MEE) at 6.5 Å^–1^ for Sr and at 5.5 Å^–1^ for Ba. To improve the comparison with the simulated spectra, the Ohta et al. methodology^57^ has been used to remove the MEEs. In the process, the spectrum range [0–10] Å^–1^ has been included for the MEE removal. In order to give a qualitative idea about the MEE impact on the EXAFS spectrum, Figure S8 in Supporting Information plots the MEE contribution to the spectrum, as resulting from the application of Ohta’s method for the case of the Ba^2+^ spectrum.^57^ As expected, the MEE contribution is larger for the heavier Ba^2+^ cation than for Sr^2+^ (not shown). As can be seen in Figure 3, there is a good agreement between the simulated EXAFS function (blue lines) and the experimental ones (green lines), once the MEE has been removed. The simulated EXAFS function has similar frequency and intensity as the experimental ones. The general theoretical–experimental agreement is a strong support for the good behavior of the developed potentials and the structural properties derived from the analysis of the MD simulations. This is also confirmed by the good agreement observed between the experimental^9^ and the simulated XANES spectra for the strontium case (see Figure 4). In their recent study on the Ba^2+^ hydration, Migliorati et al.^20^ have obtained a fairly good agreement between their experimental and simulated EXAFS spectra, with *R*_Ba–O(I)_ and *R*_Ba–H(I)_ structural parameters close to those found here. (Maxima of Ba–O_I_ and Ba–H_I_ RDFs are 2.78 vs 2.80^20^ Å and 3.35 vs 3.46^20^ Å, respectively). The main difference is the coordination number: 9.4 in our case, larger than 8.1 found by D’Angelo’s group. Nevertheless, the good theoretical–experimental agreement obtained by D’Angelo’s group as well as by us is justified by the DW-coordination number correlation, as we obtained in our simulation a higher DW factor with a larger coordination number.

**Figure 5:**
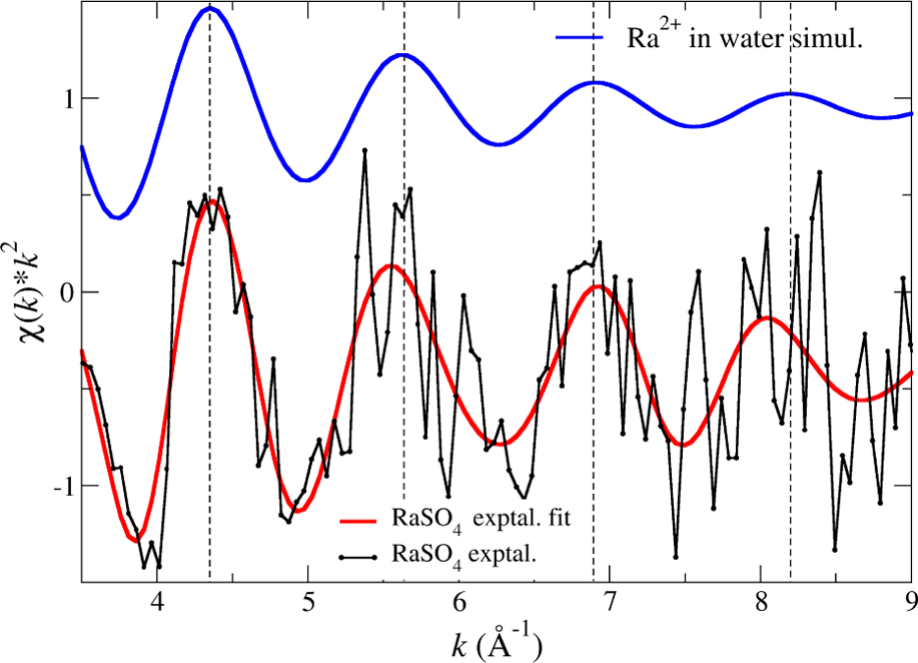
Ra L_3_-edge *k*^2^-weighted simulated EXAFS spectrum of a Ra^2+^ aqueous solution (blue line) and experimental (black line) and fitted (red line) spectrum of a RaSO_4_ crystalline sample taken from ref (25).

To our knowledge, there is no experimental EXAFS spectrum of a radium salt in water. Based on the good results obtained for the other two heavy alkaline-earth cations, we have simulated the Ra L_3_-edge *k*^2^-weighted EXAFS spectrum of a Ra^2+^ dilute aqueous solution (see blue line in Figure 5). The only EXAFS spectrum of a Ra-containing sample with Ra–O backscattering paths has been measured by Hedström et al.^25^ It corresponds to a solid sample of radium sulfate (black line in Figure 5). The fitting of this spectrum (red line) gives a coordination number of 12 and a Ra–O distance of 2.96 Å, with a DW factor for this shell of 0.007 Å^2^. The comparison between this experimental fitting and our simulated spectrum of the Ra^2+^ aqueous solution (blue line) shows a slight higher frequency in the radium sulfate case, a higher intensity, and a slower decaying of the signal at high *k*. All these features are consistent with the structural data obtained from our MD simulation: the Ra–O_H2O_, 2.93 Å, is shorter than that of Ra–O_SO4_, the coordination number, 9.8, is smaller than that of radium sulfate, and the DW factor, 0.034 Å^2^, is larger than that of the solid sample. The similarities and differences between the EXAFS spectra, corresponding to two different, but related samples, seem to suggest that this proposal of EXAFS spectrum for a Ra^2+^ dilute aqueous solution is sound.

The nonphase-shift-corrected Fourier transforms of the simulated EXAFS spectra of the three cations in water have been plotted in Figure S9 of Supporting Information. As expected, the main peak of the FT is shifted toward higher *R* values when going from Sr^2+^ to Ra^2+^. In the Ra^2+^ case, the high-*R* region shows an asymmetric shape of the peak, decaying slowly with *R*.

**Figure 6:**
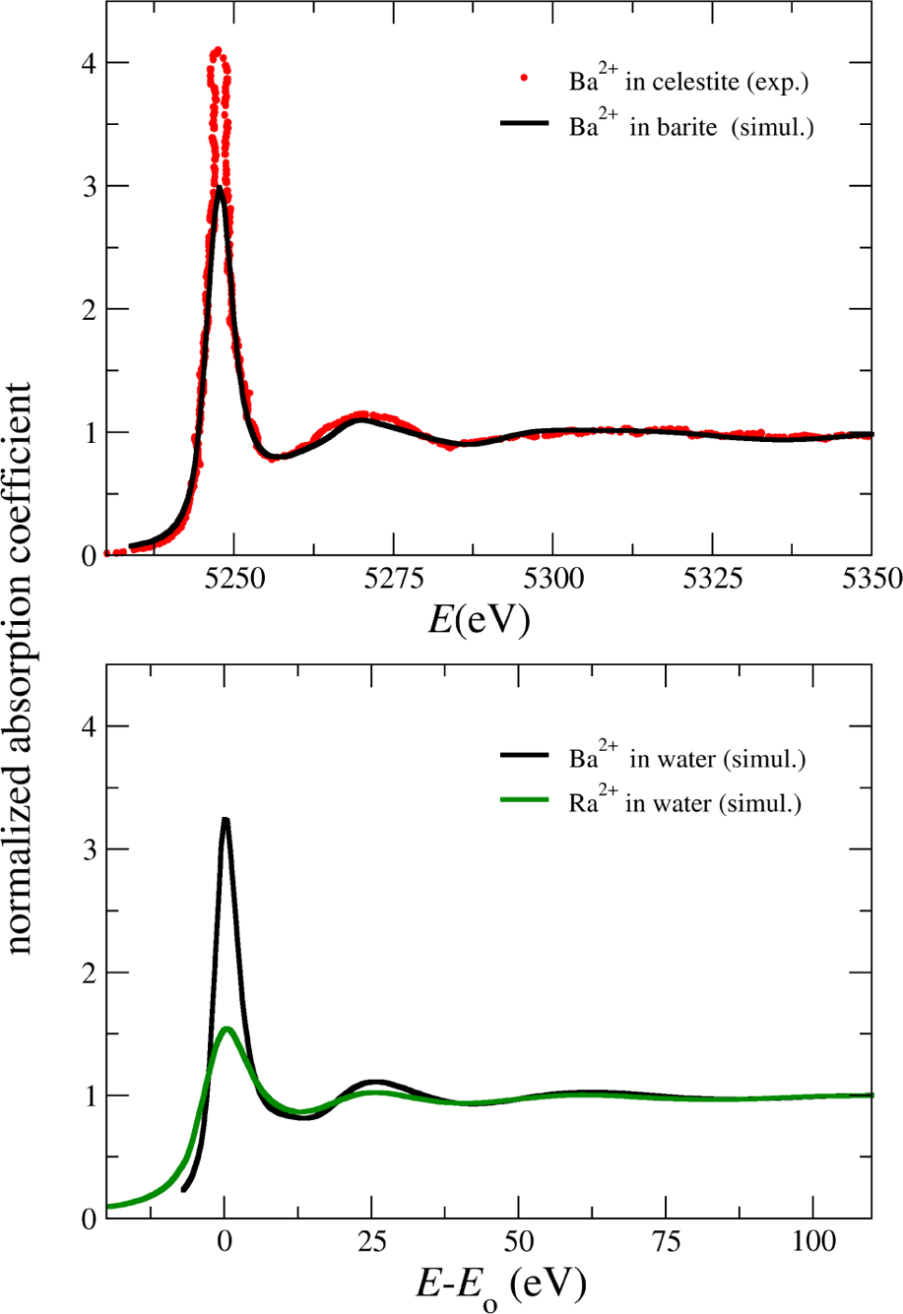
(Top) Ba L_3_-edge XANES spectrum of celestite measured by Finch et al.^64^ and the simulated spectrum taken from the Ba^2+^ cation and its closest environment from the barite. (Bottom) Ba and Ra L_3_-edges XANES simulated spectra of a Ba^2+^ and Ra^2+^ aqueous solution computed from MD snapshots.

As expected, no experimental XANES spectrum of a Ra^2+^ aqueous solution has been reported yet. Similarly, as far as we know, no XANES spectrum has been published for Ba^2+^-containing aqueous solutions. In order to get some experimental reference of a chemically related system to Ba^2+^ aqueous solution, we have examined the Ba L_3_-edge XANES spectrum of the celestite mineral (SrSO_4_) with a very low concentration of Ba (∼100 ppm) substituting Sr, as recorded by Finch et al.^64^ (red points in the top of Figure 6). This supplies a structure of 12 sulfate oxygen atoms surrounding the barium cation. The top of Figure 6 shows the comparison of this experimental spectrum (red dots) and the simulated one (black solid line) performed using the cluster of a Ba and its closest environment of sulphate anions at the experimental geometry of the isostructural barite mineral. The white line intensity is quite different, but the general shape of both spectra is similar. It must borne in mind that the simulated spectrum was not built with the same structure of the celestite but with a related structure. In any case, results are quite reasonable and give some confidence on the ability of the FEFF code to simulate the XANES spectrum of Ba^2+^ in water, where the dominant path is also Ba–O. The average XANES spectrum derived from a set of snapshots of our MD simulations of Ba^2+^ in water is displayed at the bottom of Figure 6. In the same figure, the Ra^2+^ L_3_-edge XANES simulated spectrum of Ra^2+^ in aqueous solution has also been displayed. It has a shape similar to the corresponding spectrum of Ba^2+^ in water, although its white line intensity is roughly a half of that of Ba^2+^. The shape of the Ba^2+^ spectrum in water is relatively simple, as observed in the case of the Ba^2+^ in the solid structure of barite (black line in the top of Figure 6).

### Dynamical Properties

**Figure 7:**
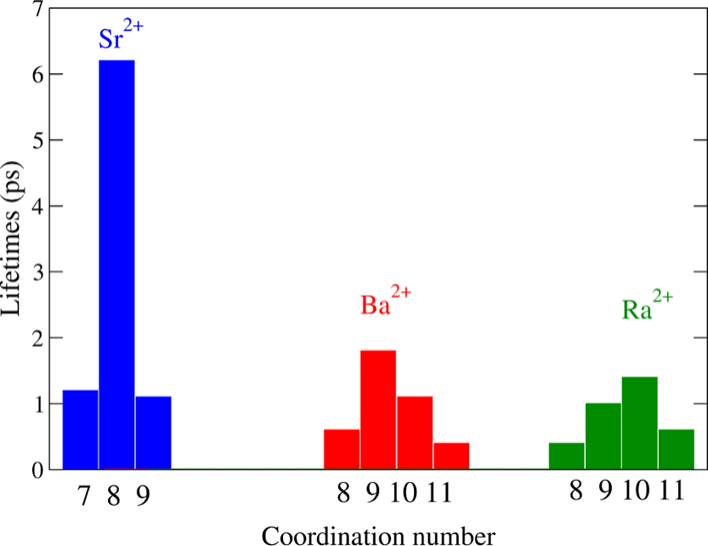
Lifetimes (ps) of different aqua ions for each heavy alkaline-earth cation.

A set of representative dynamical properties of the three cations in solution have also been collected in Table 2. In addition, the persistence of the different aqua ions as a function of their coordination number has been plotted in Figure 7 and the hydration number evolution around the metal cation as a function of time in Figures S10–S12 of Supporting Information.

Let us start by analyzing the lifetimes of the different aqua ions, a complementary information to that of the distribution of aqua ion coordination number shown in Figure 2. To emphasize this difference between the structural and dynamical information, we have designed the plot showing the time of persistence of each aqua ion of a given coordination number (Figure 7) in a way similar to that adopted for showing the distribution of the coordination number. The joint analysis of both figures clearly shows that the prevalence of a given hydrate does not guarantee a long lifetime. This is particularly clear for the cases of Ba^2+^ and Ra^2+^ aqua ions. Although there are several water exchanges for the Sr^2+^ during the simulation that changes the coordination number (see Figure S10 in Supporting Information), the lifetime of the enneahydrate and heptahydrate are rather short (∼1 ps) compared to that of the octahydrate (∼6 ps), as seen in Figure 7. For Ba^2+^, the coordination number changes between 9 and 10 with high frequency (Figure S11 of Supporting Information), their lifetimes being short, 2 and 1 ps, respectively. For Ra^2+^, the coordination number distribution is shifted to the deca-coordination (Figure 2), and this aqua ion has the longest, but still short, persistence among the different Ra^2+^ hydrates.

The dynamical behavior of the aqua ions can be also quantified by the mean residence times (MRTs) of water molecules in their first hydration shell. In this way, the values decrease along the group from strontium to radium (see Table 2). For t* = 0, 2 ps, the values are 65, 90 ps, 38, 53 ps, and 20, 29 ps for Sr^2+^, Ba^2+^, and Ra^2+^, respectively. If one bears in mind that for pure water, the MCDHO2 potential gives a value of 2.2 ps for the persistence of a water molecule in the hydration shell of another water molecule, one must recognize the ability of the divalent cation to slow down the dynamical effects of water molecules in its closest environment. This is reinforced by the analysis of their heavy alkaline partners, Rb^+^ and Cs^+^, for whose first hydration shells, the MRTs of water molecules are in the order of 6–11 ps.^42^ The comparison of the dynamical properties for the three heavy alkaline-earth cations shows how the Sr^2+^ octahydrate is also the least labile aqua ion from the dynamical view.

The cation self-diffusion coefficient (see Table 2) follows the experimental^2^ trend, increasing along the group. The higher stability of the Sr^2+^ octahydrate and its smaller size favors a strong interaction with the second hydration shell, which hampers the mobility of the rather rigid hydrated ion. The larger size of the heavier Ba^2+^ and Ra^2+^ and their more labile structures reduce the interaction with the second hydration, such as a higher cation mobility is permitted. This trend is also confirmed when analyzing the mobility of the monovalent heavy alkaline Rb^+^ and Cs^+^. Their diffusion coefficients are ∼1.2 × 10^–5^ cm^2^ s^–1^ values higher than those of the alkaline-earth cations studied (0.6–0.9 × 10^–5^ cm^2^ s^–1^).

The reorientational dynamics of the first-shell water molecules is similar for Ba^2+^ and Ra^2+^ aqua ions and is slower for those water molecules surrounding Sr^2+^. Table S3 in Supporting Information collects the values for the first- (*i* = 1) and second-order (*i* = 2) reorientational times around the dipole moment (τ_*i*,μ_), the H–H and O–H axis (τ_*i*,H–H_; τ_*i*,O–H_), and the normal direction to the molecular plane (τ_*i*,_⊥). These results correlate well with the previous dynamical discussion. For all the ions, the dynamics of the first-shell water molecules is slower than in bulk water. The intrinsic dynamics of water molecules forming the first hydration shell is more restricted in their orientational mobility around the cation for the Sr^2+^ case, which once more confirms the stability of its aqua ion. The orientational dynamics of the Ba^2+^ and Ra^2+^ water molecules is similar and points out the more labile character of their hydrates.

## Concluding Remarks

The behavior of the intermolecular potentials developed to describe the hydration of the heaviest alkaline-earth cations in water by classical MD simulations has been shown to be satisfactory. Energy, structural, dynamical, and spectroscopical properties derived from the analysis of the statistical trajectories are in reasonable agreement with the available experimental information. The scarce information on Ra^2+^ hydration makes the assessment about this cation more difficult, but the good results for the other two cations are a convincing support as the three cations were treated on the same methodological foot to derive their intermolecular potentials. Likewise, indirect comparisons with chemically related samples gives confidence of the predicted theoretical results. The good behavior of the Ra^2+^ potential offers an additional route toward the computation of a wide range of thermodynamic properties of solutions containing its hydrated ions at moderate computational efforts using classical MD simulations. This could help to provide a new view on the often contradicting results found in the literature. The comparison of the results obtained provides a picture, where the Ba^2+^ and Ra^2+^ cations show similar hydration properties. Their hydrates exhibit a significant flexibility and ability to change their large hydration shells; thus, we may consider that the Sr^2+^ aqua ion behavior differs from theirs because it appears like a more stable and consistent hydrated complex, that is a stable aqua ion. The comparison between the heavy alkaline-earth and alkaline cations leads to conclude that for the former ions, the ion–solvent interactions are dominant with respect to the solvent–solvent ones. This determines their structural and dynamical properties: Ba^2+^ and Ra^2+^ are mild aqua ions.

On the basis of the results obtained here, Ba^2+^ is a good candidate to substitute Ra^2+^ in experimental efforts devoted to get insights into the control mechanisms of Ra^2+^ release from the nuclear waste into aquifers and further related processes.^65,66^

## Supporting Information Available

Parameter force field obtained for M^2+^–H_2_O (M = Sr, Ba, Ra), representative set of structure types employed in the fitted potentials, plots showing the correlation between the QM interaction energies and the predicted values by the developed force field, examples of input FEFF files used for the XAS spectrum simulations, MEE contribution to the Ba EXAFS spectrum, FT modulus of the simulated spectra of Sr^2+^, Ba^2+^, and Ra^2+^ in water, and the time evolution of the cation first-shell hydration numbers (PDF)
