# Supplementary material for: Hydration of Heavy Alkaline-Earth Cations Studied by Molecular Dynamics Simulations and X-ray Absorption Spectroscopy
Source: Inorg Chem. 2021 Aug 13;60(17):13578–87. doi: 10.1021/acs.inorgchem.1c01888 (PMC8512670; doi:10.1021/acs.inorgchem.1c01888)
Supplement: Supplementary file 1 — ic1c01888_si_001.pdf [file ic1c01888_si_001.pdf]

Supporting Information

Hydration of Heavy Alkaline-Earth Cations Studied by Molecular Dynamics Simulations and X-ray Absorption Spectroscopy

Rafael R. Pappalardo, Daniel Z. Caralampio, José M. Martínez, and Enrique Sánchez Marcos*

Universidad of Sevilla, Departmento de Química Física, 41012 Sevilla, (Spain).

e-mail:sanchez@us.es

Intermolecular ion-water potential and MCDHO2 water potential

The monoatomic cation, M(II), (M being Sr, Ba or Ra) is described by a positive charge, $Z_{\rm M} = 5$, and a mobile negative charge density, $\rho_{\rm M}$, with a total charge $q_{\rm M} = -3$, joined to the nucleus by a spring of force constant, $k_{\rm M}$(see Figure S1). The intra-atomic energy is defined by:

$$U_{intra} = \frac{1}{2} k_{\rm M} \cdot r^2 \tag{1}$$

where r is the distance between the nucleus and its associated mobile charge density. In the absence of an external field, the equilibrium position of the oscillator is located on the nucleus and $U_{intra} = 0$.

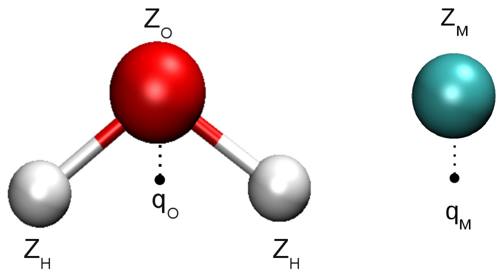

Figure S1: Schematic representation of MCDHO2 model for the water molecule and the metal cation.

The value of $k_{\rm M}$ together with the $q_{\rm M}$ value of the mobile charge defines the polarizability by the equation $\alpha = \frac{q_{\rm M}^2}{k_{\rm M}}$. For Sr^{2+} and Ba^{2+} the experimental polarizability is reproduced. There is not such information for Ra^{2+}, but theoretical estimations for neutral Ra show that its value is slightly smaller than that of Ba. This is due to relativistic effects, as observed in the case of the alkaline group, when passing from Cs to Fr. (Schwerdtfeger, P. and Nagle, J.K. *Mol. Phys.* **2019**, *117*, 1200-1225.) In the case of the divalent cations, the polarizability decreases about ten times and the relativistic effects are no as important as for the neutral atoms. (Lim, S. and Schwerdtfeger, P. *Phys. Rev. A* **2004**, *70*, 062501.) On this basis we decided to impose the same $k_{\rm M}$ for the Ba^{2+} and Ra^{2+} ions.

The incorporation of the MCDHO2 model to the new potential needs the consideration of the following intermolecular terms for M(II)-H_2O interaction:

- Classical interaction between the mobile charge densities, $q_{\rm O}$ and $q_{\rm M}$, given by a two-exponential function:

$$U_{inter}(q_{\rm O}, q_{\rm M}) = A_{\rm MO} \cdot e^{-\alpha_{\rm MO} \cdot r_{\rm MO}} + B_{\rm MO} \cdot e^{-\beta_{\rm MO} \cdot r_{\rm MO}} \tag{2}$$

  where $r_{\rm MO}$ is the distance between the mobile charge densities. $A_{\rm MO}$, $\alpha_{\rm MO}$, $B_{\rm MO}$ and $\beta_{\rm MO}$ are fitting parameters.

- Classical interaction between the M nucleus, $Z_{\rm M}$, and those of the water molecule, $Z_i (i \equiv$O, H), is given by a two-exponential function as well:

$$U_{inter}(Z_i, Z_{\rm M}) = C_{{\rm M}i} \cdot e^{-\gamma_{{\rm M}i} \cdot R_i} + D_{{\rm M}i} \cdot e^{-\delta_{{\rm M}i} \cdot R_i} \tag{3}$$

  where R_i is the distance between the M nucleus and each i-th nucleus of the water molecule, and $C_{{\rm M}i}$, $\gamma_{{\rm M}i}$, $D_{{\rm M}i}$ and $\delta_{{\rm M}i}$ are fitting parameters.

- Electrostatic interaction between the water mobile charge density, $q_{\rm O}$ and the M nucleus, $Z_{\rm M}$:

$$U_{inter}(q_{\rm O}, Z_{\rm M}) = \frac{q_{\rm O} Z_{\rm M}}{r'} \left[1 - \left(\frac{r'}{\lambda'} + 1 \right) e^{-2r'/\lambda'} \right] \tag{4}$$

  where r' is the distance between the center of $\rho_{\rm O}$ and the M nucleus and λ' is the intermolecular screening described in the original MCDHO2 paper (Villa, A.; Hess, B.; Saint-Martin, H. J. Phys. Chem. B **2009**, *113*, 7270-7281).

- Electrostatic interaction between the M mobile charge density, $q_{\rm M}$, and each of the charges on the water molecule nuclei, $Z_i (i \equiv$O, H):

$$U_{inter}(Z_i, q_{\rm M}) = \frac{Z_i {\rm q_M}}{r_i}\left[1 - \left(\frac{r_i}{\lambda'_{\rm M}} + 1\right) e^{-2r_i/\lambda'_{\rm M}}\right] \tag{5}$$

where r_i is the distance from the $\rho_{\rm M}$ center to Z_i and $\lambda'_{\rm M}$ is the corresponding intermolecular screening.

Thus, the interaction energy for a cluster with N water molecules is computed by the expression:

$$\begin{aligned} U = \sum_{S=1}^{N} \Bigg(\sum_{i\in S}\sum_{j\in T} [U_{inter}(Z_i, Z_j) + U_{inter}(q_i, q_j) \\ + U_{inter}(q_i, Z_j) + U_{inter}(q_j, Z_i)] + \sum_{i\in S} \frac{1}{2} k_i \cdot r_{ii}^2 + \frac{1}{2} k_{\rm M} \cdot r^2 \Bigg) \end{aligned} \tag{6}$$

where S runs over the water molecules and T over the M.

The MCDHO2 water molecules are described by a positive charge in the hydrogens $Z_{\rm H} = 0.62$ and in the oxygen $Z_{\rm O} = 2.00$ and with a negative charge in the mobile charge density $q_{\rm M} = -3.24$ joined to the oxygen by a spring of force constant, k(see Figure S1) and with a fictitious mass of 0.1 a.u.:

$$U_k = \frac{1}{2} k \cdot r^2 \tag{7}$$

The charge density is modeled by λ:

$$\rho(r) = \frac{q}{\pi\lambda^3} {\rm e}^{-2\frac{r}{\lambda}} \tag{8}$$

being the charge:

$$q = 4\pi \int_0^\infty \rho(r) r^2 dr \tag{9}$$

The interatomic distance is modeled by a Morse potential:

$$U_{d_{\rm OH}} = D_{\rm OH}\left({\rm e}^{-2\gamma(R_\beta - r_{\rm e})} - 2{\rm e}^{-\gamma(R_\beta - r_{\rm e})}\right) \tag{10}$$

The internal angle is defined by a quartic potential:

$$U_{\Theta_{\rm HOH}} = {\rm a}_1(\Theta - \Theta_{\rm e}) + {\rm a}_2(\Theta - \Theta_{\rm e})^2 + {\rm a}_3(\Theta - \Theta_{\rm e})^3 + {\rm a}_4(\Theta - \Theta_{\rm e})^4 \tag{11}$$

Being the internal energy of a water molecule:

$$U_{\rm internal} = \frac{1}{2} k r_{\rm O}^2 + \frac{Z_{\rm H}^2}{R_{1,2}} + \frac{q Z_{\rm H}}{r_\beta}\left[1 - \left(\frac{r_\beta}{\lambda} + 1\right) e^{-2r_\beta/\lambda}\right] + U_k + U_{d_{\rm OH}} + U_{\Theta_{\rm HOH}} \tag{12}$$

The energy of a cluster of N water molecules is defined by a Lennard-Jones potential for the interactions between oxygens, between hydrogens and between oxygen and hydrogen.

$$\begin{aligned} U_{\rm total} = \sum_{n=1}^{N}\sum_{m=1}^{n-1} \left(\frac{A}{r_{nm}}\right)^{12} - \left(\frac{B}{r_{nm}}\right)^{6} + \frac{q^2}{r_{nm}} + \frac{q Z_\beta}{r_{n\beta}}\left[1 - \left(\frac{r_{n\beta}}{\lambda} + 1\right) e^{-2r_{n\beta}/\lambda}\right] \\ + \sum_{\beta\in m}^{q} \left(\frac{A_{\alpha\beta}}{r_{\alpha\beta}}\right)^{12} - \left(\frac{B_{\alpha\beta}}{r_{\beta\alpha}}\right)^{6} + \frac{Z_\alpha \beta}{r_{\alpha\beta}} \end{aligned} \tag{13}$$

Table S1: Parameters of the MCDHO2 water potential (a.u).

Z_{H}	0.62
Z_{O}	2.0
q	-3.24
k	1.00
λ	1.90
D_{OH}	0.42954902
r_e	1.3440633
γ	1.1131102
θ_e	1.927
a_1	0.031621
a_2	0.043914
a_3	-0,012721
a_4	-0.00866
A_{OM}	3.228656
B_{OM}	1.962046
A_{OH}	2.037891
A_{HH}	0

Table S2: Fitted parameters (a.u.) of the alkaline-earth potentials.

	$\mathbf{Sr}^{2+}$	$\mathbf{Ba}^{2+}$	$\mathbf{Ra}^{2+}$
k_{M}	1.269662	0.650790	0.650790
λ'_{M}	0.414399	0.435619	0.554093
A_{MO}	277.432810	205.594587	248.886579
α_{MO}	1.560342	1.445294	1.474152
B_{MO}	-162.291509	-88.434493	-88.710280
β_{MO}	1.454231	1.289500	1.294386
C_{MH}	53.495873	79.272175	105.444582
γ_{MH}	2.603508	3.226664	2.628450
D_{MH}	0.000038	0.000048	0.000045
δ_{MH}	1.415779	1.574448	1.256433

Table S3: First, $\tau_{1,n}$, and second order, $\tau_{2,n}$, reorientational times of first shell water molecules.

	τ (ps)							
Ion	$\tau_{1,\mu}$	$\tau_{2,\mu}$	$\tau_{1,\mathrm{HH}}$	$\tau_{2,\mathrm{HH}}$	$\tau_{1,\perp}$	$\tau_{2,\perp}$	$\tau_{1,\mathrm{OH}}$	$\tau_{2,\mathrm{OH}}$
Sr^{2+}	44	8	7	5	5	2	16	4
Ba^{2+}	26	4	6	4	5	2	10	3
Ra^{2+}	23	4	5	4	4	2	11	3
Bulk	5	2	6	3	4	2	6	3

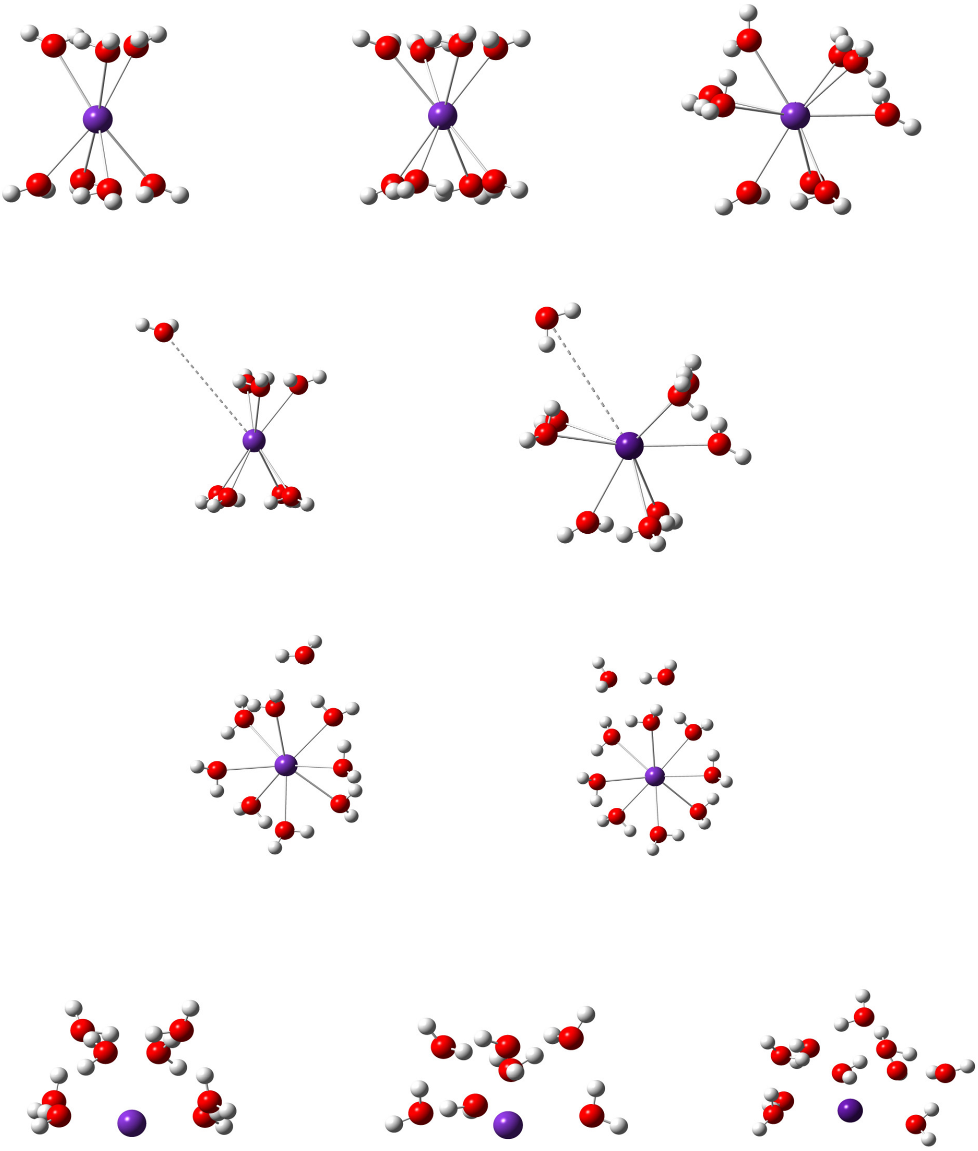

Figure S2: Representative structures of alkaline-earth hydrates employed in the development of the metal ion-water potential.

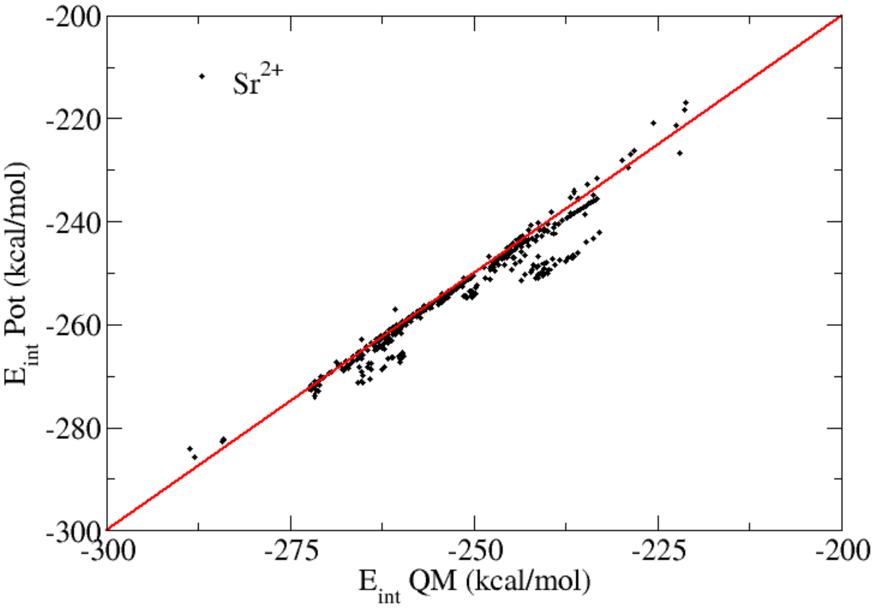

Figure S3: Sr^{2+}-H_2O fitting: Interaction energy derived from potential vs QM energy of all structures used for the fitting.

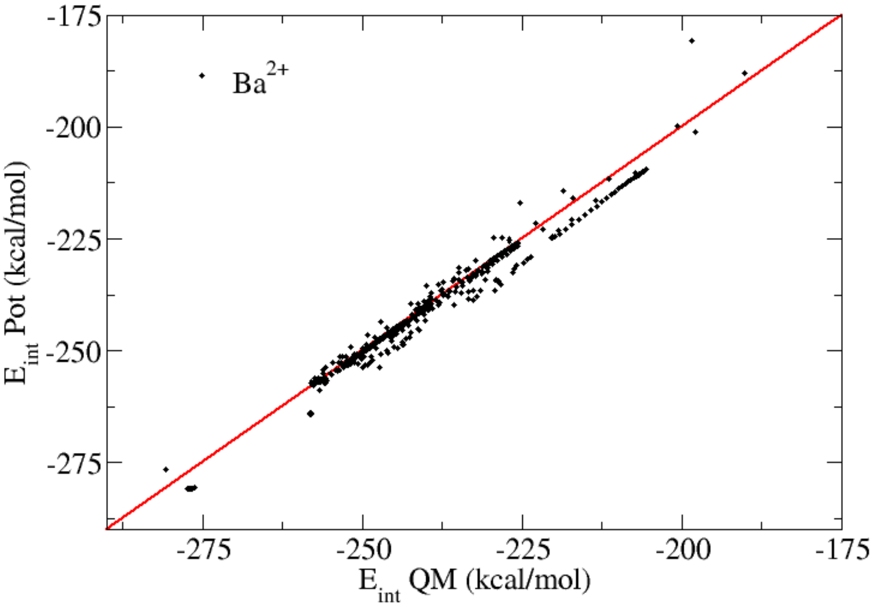

Figure S4: Ba^{2+}-H_2O fitting: Interaction energy derived from potential vs QM energy of all structures used for the fitting.

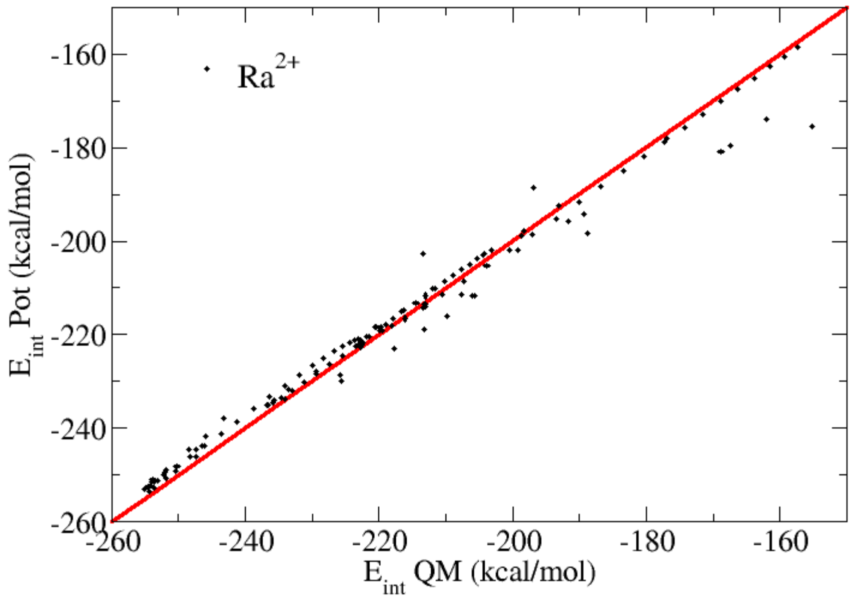

Figure S5: Ra^{2+}-H_2O fitting: Interaction energy derived from potential vs QM energy of all structures used for the fitting.

```
EDGE K
CONTROL 1 1 1 1 1 1
PRINT 0 0 3 0 0 0
COREHOLE RPA
EXAFS 10.0
CRITERIA 4.0 2.5
RPATH 6.0
NLEG 4
TDLDA  1
SCF 6.0 0
EXCHANGE 0 -3.0 0.0 2
POTENTIAL
       0    38   Sr     3   3
       1    8    O     3   3
       2    1    H     2   2


ATOMS
   0.0000000    0.0000000    0.0000000  0  Sr    0.0000000
  -0.5510200    1.9859500   -1.3270000  1  O    2.4512343
  -1.4491040    2.3771500   -1.2250000  2  H    3.0416064
  -0.1454000    2.2630500   -2.1810000  2  H    3.1463149
  -0.0122000   -2.4482500   -0.2110000  1  O    2.4573559
  -0.4844600   -2.9217500    0.4780000  2  H    2.9999681
   0.1347000   -3.1290500   -0.8850000  2  H    3.2545849
  -2.3995000    0.3135900    0.5480000  1  O    2.4811777
  -2.8195000   -0.4639300    0.1100000  2  H    2.8595299
  -3.0853000    0.9797500    0.6510000  2  H    3.3019369
   2.4778000   -0.2722510    0.2300000  1  O    2.5033005
   2.7141000   -0.6693600    1.1200000  2  H    3.0114418
   2.8367000   -0.9735700   -0.3700000  2  H    3.0218546
  -1.3136100   -0.7790000   -2.0980000  1  O    2.5949983
  -0.6223700   -1.0413300   -2.7700000  2  H    3.0240060
  -2.0930600   -0.4296400   -2.6390000  2  H    3.3955576
  -0.1704000    1.7875500    1.8938000  1  O    2.6097604
  -0.9250200    2.3334500    2.2801000  2  H    3.3910923
   0.4696000    1.7077500    2.6028000  2  H    3.1482538
  -0.5658400   -1.1575300    2.3635000  1  O    2.6918735
  -0.4974900   -0.4054900    2.9855000  2  H    3.0537074
  -1.5022240   -1.3683500    2.4805000  2  H    3.2065463
   1.3524000    0.1437700   -2.3390000  1  O    2.7056564
   1.0505000    0.6123200   -3.1410000  2  H    3.3681400
   2.3693000    0.1677900   -2.3540000  2  H    3.3441071
END
```

Figure S6: FEFF input file for computation of simulated EXAFS spectrum of Sr^{2+} in water

```
EDGE K

CONTROL 1 1 1 1 1 1

PRINT 0 0 3 0 0 0

XANES
FMS 6.0 1
AFOLP
OPCONS
MPSE 2
COREHOLE RPA
TDLDA  1
SCF 6.0 0
EXCHANGE 0 0.0 0.0 2
POTENTIAL
     0   38   Sr    3   3
     1    8    O    3   3
     2    1    H    2   2


ATOMS
  0.0000000   0.0000000   0.0000000  0  Sr   0.0000000
 -0.5510200   1.9859500  -1.3270000  1  O    2.4512343
 -1.4491040   2.3771500  -1.2250000  2  H    3.0416064
 -0.1454000   2.2630500  -2.1810000  2  H    3.1463149
 -0.0122000  -2.4482500  -0.2110000  1  O    2.4573559
 -0.4844600  -2.9217500   0.4780000  2  H    2.9999681
  0.1347000  -3.1290500  -0.8850000  2  H    3.2545849
 -2.3995000   0.3135900   0.5480000  1  O    2.4811777
 -2.8195000  -0.4639300   0.1100000  2  H    2.8595299
 -3.0853000   0.9797500   0.6510000  2  H    3.3019369
  2.4778000  -0.2722510   0.2300000  1  O    2.5033005
  2.7141000  -0.6693600   1.1200000  2  H    3.0114418
  2.8367000  -0.9735700  -0.3700000  2  H    3.0218546
 -1.3136100  -0.7790000  -2.0980000  1  O    2.5949983
 -0.6223700  -1.0413300  -2.7700000  2  H    3.0240060
 -2.0930600  -0.4296400  -2.6390000  2  H    3.3955576
 -0.1704000   1.7875500   1.8938000  1  O    2.6097604
 -0.9250200   2.3334500   2.2801000  2  H    3.3910923
  0.4696000   1.7077500   2.6028000  2  H    3.1482538
 -0.5658400  -1.1575300   2.3635000  1  O    2.6918735
 -0.4974900  -0.4054900   2.9855000  2  H    3.0537074
 -1.5022240  -1.3683500   2.4805000  2  H    3.2065463
  1.3524000   0.1437700  -2.3390000  1  O    2.7056564
  1.0505000   0.6123200  -3.1410000  2  H    3.3681400
  2.3693000   0.1677900  -2.3540000  2  H    3.3441071
END
```

Figure S7: FEFF input file for computation of simulated XANES spectrum of Sr^{2+} in water

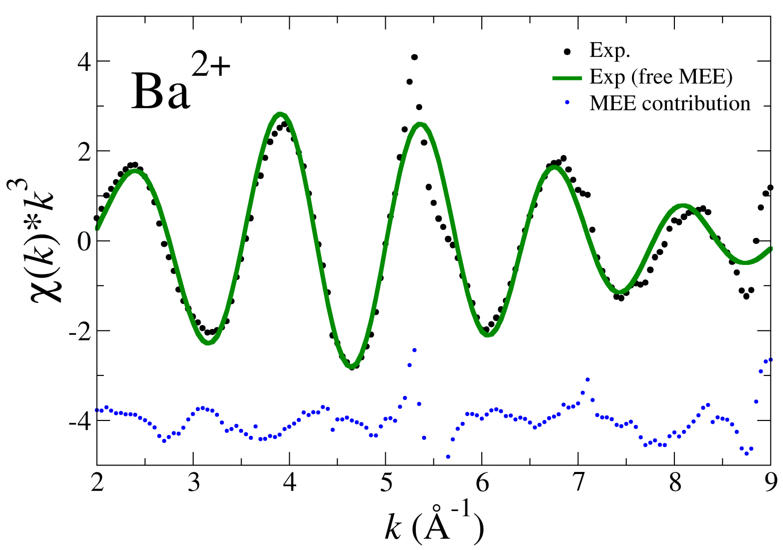

Figure S8: EXAFS spectrum of Ba with (black dotted line) and without (green line) MEE and the MEE contribution (blue dotted line).

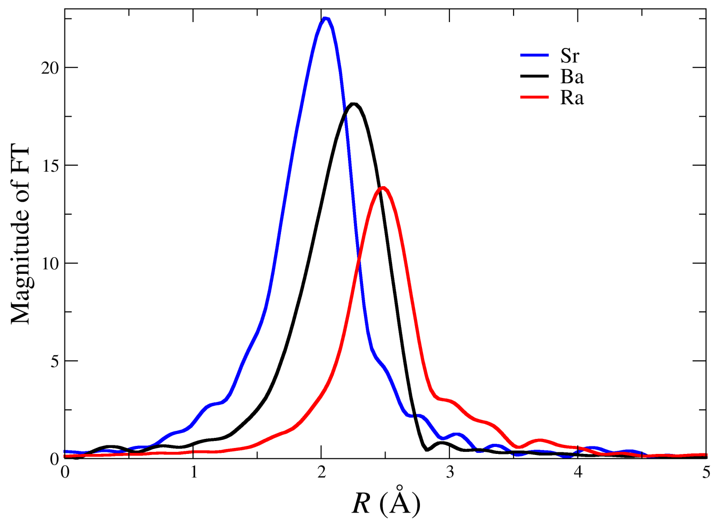

Figure S9: Nonphase-shift-corrected Fourier transforms of the simulated spectra of Sr^{2+}, Ba^{2+} and Ra^{2+} in water.

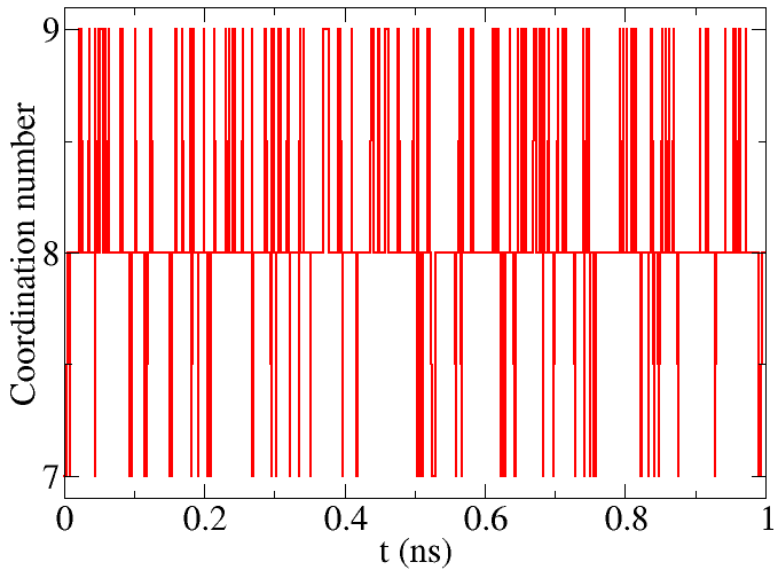

Figure S10: Time evolution of the Sr^{2+} coordination number in aqueous solution.

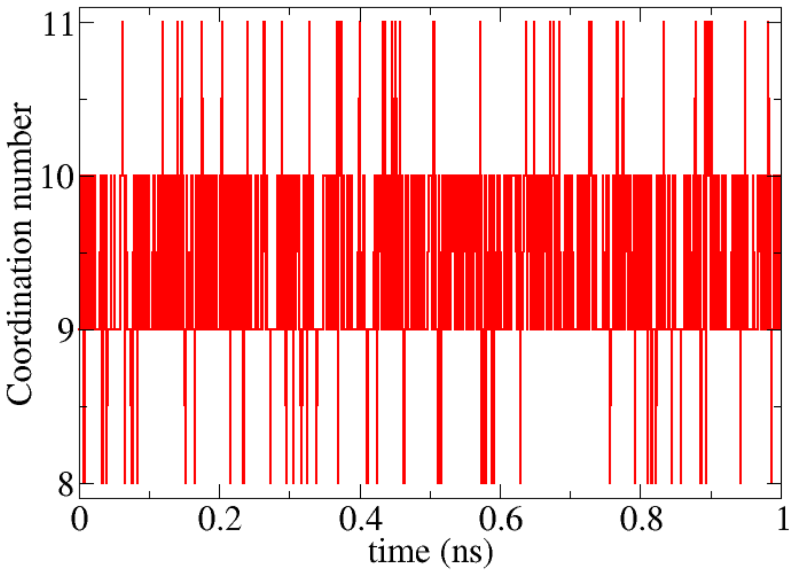

Figure S11: Time evolution of the Ba^{2+} coordination number in aqueous solution.

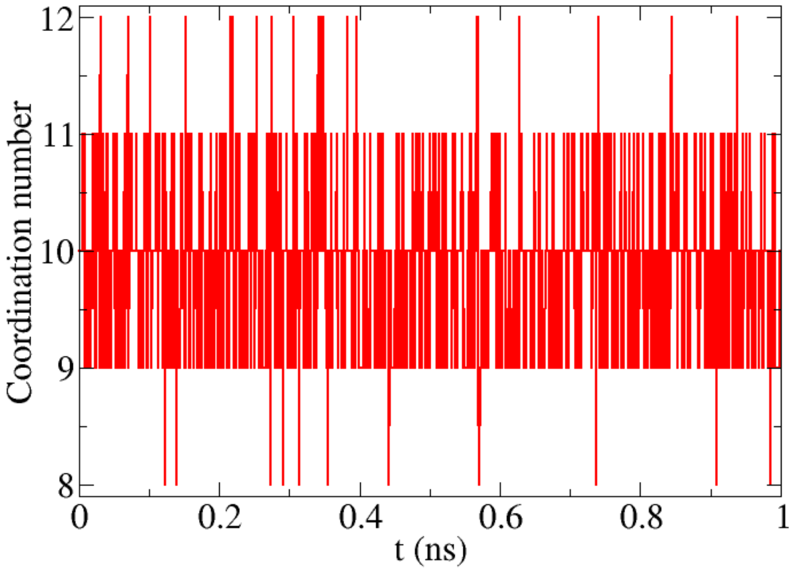

Figure S12: Time evolution of the Ra^{2+} coordination number in aqueous solution.
